# Comparison of prevalence of diabetes complications in Brazilian and Mexican adults: a cross-sectional study

**DOI:** 10.1186/s12902-021-00711-y

**Published:** 2021-03-16

**Authors:** Renata Breda Martins, Sandra Azucena Ordaz-Briseño, Sarahí Flores-Hernández, Ângelo José Gonçalves Bós, Raúl C. Baptista-Rosas, Arieh Roldán Mercado-Sesma

**Affiliations:** 1 Biomedical Gerontology at Pontifical Catholic University of Rio Grande do Sul, Porto Alegre, Rio Grande do Sul Brazil; 2 Centro Universitario de Tonalá, Universidad de Guadalajara, Guadalajara, Mexico; 3 Departamento de Salud Enfermedad como proceso individual, Centro Universitario de Tonalá, Universidad de Guadalajara, Tonalá, Jalisco Mexico; 4 Multidisciplinary Health Research Center, Centro Universitario de Tonalá/Universidad de Guadalajara (México), 45425 Tonalá, Jalisco Mexico

**Keywords:** Diabetes complications, Diabetes risk, Health surveys, Diabetes complications prevalence

## Abstract

**Background:**

Type 2 diabetes is more frequent in Latin American people than in non-Hispanic whites due to a combination of genetic and lifestyle risk factors. Brazil and Mexico are the most populous countries in Latin America. The present study aimed to compare the results of the National Health Survey “PNS” in Brazil and the National Survey Health and Nutrition “ENSANUT” in Mexico regarding the prevalence, complications and healthcare issues of diabetes in both countries.

**Methods:**

A cross-sectional study was conducted with data from the National Health Survey (PNS) of 2013 in Brazil and the National Survey of Health and Nutrition (ENSANUT) of 2018 in Mexico. The prevalence of diabetes, complications and risk factors related to developing diabetes were considered.

**Results:**

The respondents included 3636 individuals in Brazil and 4555 individuals in Mexico. There were significant differences in age and time living with diabetes between the two countries. Mexican people had twice as likely as Brazilian people to have a complication (*p* < 0.0001). The principal risk factor (OR 2.47; *p* ≤ 0.0001) for developing any diabetic complication was living with diabetes for more than 15 years. Visual impairment was the most frequent complication in both countries, but it was more prevalent in Mexico (*p* ≤ 0.001).

**Conclusions:**

Diabetes complications are important health problems in Brazil and Mexico. Visual impairment was the principal complication in both countries. Several factors, such as access to and type of health system, living in a rural area, treatment, BMI and performing preventive actions, affected the risk of developing a complication. However, living with diabetes for more than 15 years was the principal risk factor. National health surveys have added significant information on the impact of diabetes in these Latin American populations. This comparison of data could provide valuable information to guide national policies and program decisions in both countries.

## Background

Diabetes is a growing global problem. The last report of the International Diabetes Federation (IDF) in 2017 estimated the global prevalence of diabetes at 424.9 million (8.8%), indicating that 1 in 11 people had diabetes [1]. Type 2 diabetes is more frequent in Latin American people than non-Hispanic whites [2] due to a combination of genetic and lifestyle risk factors [3]. Brazil and Mexico are the most populous countries in Latin America [4]. The age-adjusted prevalence of diabetes in Brazil is 8.7%, compared to 14.7% in Mexico. This estimation classifies Brazil as the country with the 3rd highest number of undiagnosed diabetics and the 5th highest number of diabetic older-adults, accounting for 4.9 million people. This number is expected to increase to 11.9 million by 2045. The same report ranks Mexico as the 8th country for undiagnosed diabetics and the 9th country for diabetic older-adults, at 4.5 million; this is expected to increase to 7.6 million people in 2045 [1].

The cost generated by diabetes in Mexico is near USD 19 billion, and it reaches USD 24 billion in Brazil. Those costs correspond to a per-patient expense of $1583 and 1920 in Mexico and Brazil, respectively [1, 5, 6].

Although laws in the constitutions of both Mexico and Brazil ensure access to health, they emphasize the issue of diabetes in both populations affected by this disease from different perspectives [7–11]. In response to this health problem, both countries performed national surveys of health in order to undertake a situational diagnostic of the population regarding their health problems, including non-communicable diseases (NCDs), such as diabetes, hypertension, dyslipidemia, and obesity, among others [12, 13]. Although the national surveys were developed independently by each country, many of their questions were similar, allowing us to compare different aspects of the prevalence and healthcare of the diseases. Although the two surveys were conducted in different years, these are the most important recent epidemiological reports in their respective countries.

The aim of the present study was to compare the results of the National Health Survey “PNS” in Brazil and the National Survey Health and Nutrition “ENSANUT” in Mexico regarding the prevalence, complications and healthcare issues of diabetes in both countries.

## Methods

A cross-sectional study was conducted with data from the National Health Survey (PNS) of 2013 in Brazil and the National Survey of Health and Nutrition (ENSANUT) of 2018 in Mexico. These are national surveys based on household sampling conducted by the Brazilian Institute of Geography and Statistics with the Ministry of Health/Brazil and the National Institute of Public Health/Mexico, respectively. We decided to compare these surveys from different years because both provide the most recent results from each country. These surveys are not conducted annually, and these reports contain the most important epidemiological information.

We included all adult (18 years and older) participants from both surveys who reported receiving a diagnosis of diabetes by a medical doctor. The present analysis excluded those participants reporting diabetes only during pregnancy.

The study protocols were approved by the ethics committee of the Centro Universitario de Tonalá of the University of Guadalajara in Mexico and the Pontifical Catholic University of Rio Grande do Sul, Brazil and were conducted according to Good Clinical Practice and the principles of the Declaration of Helsinki. The report considered the STROBE statement.

### Materials and variables

The variables used in PNS were taken from modules P (lifestyles) and Q (chronic diseases). In the case of ENSANUT, the variables of section III (diabetes mellitus) and XIII (risk factors) were considered. After comparison with a X^2^ test, all variables that were significant (*p* < 0.05) were included in the logistic regression analysis. These variables were sex (female/male); age (years); area of residence (rural/urban); use of alcohol (yes/no); diagnosis of diabetes made by a physician (yes/no); time living with diabetes (years); type of diabetes treatment (none/oral medications/insulin/both); if they engaged in physical activity (yes/no); BMI based on silhouette images; diagnosis of cardiovascular disease (yes/no) or dyslipidemia (yes/no); access to medical care (yes/no); type of medical care (public/private/do not receive/other); indicators of glycemic control, such as measurements of venous glucose (yes/no) and Hb_A1C_ (yes/no) and use of glucose strips (yes/no); preventive measures, such as urinalysis (yes/no) and foot exam (yes/no); and complications due to diabetes (amputation/coma/renal impairment/leg ulcers/myocardial infarction/visual impairment). All the data were obtained through a direct question of whether the participant had been diagnosed with any of these complications during the last year.

For the purposes of statistical analysis, some variables had their responses recategorized. The duration of living with diabetes was categorized as < 5 years, 5 to < 10 years, 10 to < 15 years, or 15 years or more. Obesity status was collected in different ways across the surveys. In Brazil, self-reported height and weight were recorded, and the body mass index was calculated in kg/m^2^. Mexico used the Stunkard scale, consisting of 9 silhouette figures that gradually increased in size from very thin (a value of 1) to very obese (a value of 9). Those results were classified as underweight (Figs. 1 and 2 and BMI, < 18.5 kg/m^2^), normal weight (Figs. 3 and 4 and BMI between 18.5 and 25 kg/m^2^), overweight (Figs. 5 through 7 and BMI between 25 and 30 kg/m^2^), and obese (Figs. 8 and 9 and BMI ≥ 30 kg/m^2^) following the classification of Bhuiyan et al. [14] The frequency of alcohol consumption was reclassified for both countries: never drinks, drinks less than once a month, and drinks once or more per month. All of the complications reported were questioned if these were present within the past year.

### Statistical analysis

The frequency distribution of sociodemographic and healthcare characteristics of diabetes and the diabetes complications for each country were computed, and their associations were tested by *Χ*^*2*^, except for age, where mean and standard deviation were calculated for each country, and the difference was tested by an unpaired Student’s t-test. The odds ratio for having any diabetes complication was calculated for all variables that were significant (*p* < 0.05) in the descriptive analyses in the two logistic regression models: simple models, with a regression performed with each variable separately, and an adjusted model, with all variables selected. All statistical analyses were conducted using SPSS software, version 25 (IBM Corporation, Armonk, NY, USA).

## Results

### Characteristics of the subjects

Diabetes was reported by 3636 Brazilians (6.8% of all *n* = 60,203 participants) and 4555 Mexicans (10.5% of *n* = 43,071). Table 1 shows the demographic characteristics of both populations and some variables used in the logistic regression. Most of the population with diabetes lived in urban areas in both countries (*p* ≤ 0.0001). In Mexico, more people overall live in rural areas than in the rural areas of Brazil (*p* = 0.001). Most people that lived with diabetes reported having less than 5 years from diagnosis in both countries (*p* ≤ 0.0001). However, a quarter of the interviewed population reported more than 15 years of living with the disease. Physical activity is a problem in both countries because most people mentioned they do not engage in physical activity (*p* ≤ 0.0001). Regarding body weight, more than half of the participants selected silhouettes related to overweight and obesity (*p* ≤ 0.0001). Both categories were more prevalent in Mexicans than Brazilian people (*p* ≤ 0.0001). More than three quarters of the people that participated in both surveys answered that they do not drink alcohol (*p* = 0.0001). Although most people indicated that they did not have cardiovascular diseases or dyslipidemia, these questions were likely misinterpreted.

**Table 1:** Demographic characteristics of diabetic participants in Brazil and Mexico

Characteristics	Countries					
	Brazil n (%) ***N*** = 3636	Mexico n (%) ***N*** = 4555	Total n (%)	***X***^***2***^	***df***	P*
**Sex**						
Men	1281 (35.3)	2751 (60.4)	4032 (49.2)	8.518	1	< 0.0001
Woman	2355 (64.7)	1804 (39.6)	4159 (50.8)			
**Age, years**	59.6 ± 13.8	58.7 ± 13.3	59.2 ± 13.5	–	–	< 0.001**
**Area of residence**						
Urban	3093 (85.0)	3444 (75.6)	6537 (79.8)	184.233	1	< 0.0001
Rural	543 (15.0)	1111 (24.4)	1654 (20.1)			
**Time living with DM**						
< 5 years	1490(40.9)	1604 (35.2)	3094(37.7)	439.226	3	< 0.0001
5 to < 10 years	665(18.2)	986 (21.6)	1651(20.1)			
10 to < 15 years	548(15.0)	645 (14.1)	1193(14.5)			
15 years or more	933(25.6)	1320 (28.9)	2253(27.5)			
**Physical activity**						
No	2827 (77.7)	3899 (85.5)	6726 (82.1)	236.380	1	< 0.0001
Yes	809 (22.2)	656 (14.4)	1465 (17.9)			
**Alcohol consumption**						
No	2837 (78.0)	3415 (74.9)	6252 (76.3)	215.464	2	< 0.0001
Less than monthly	324 (8.9)	407 (8.9)	731 (8.9)			
At least monthly	475 (13.0)	733 (16.0)	1208 (14.7)			
**Silhouette**						
Underweight	306 (8.4)	469 (10.2)	495 (6.0)	1099.902	3	< 0.0001
Normal weight	576 (15.8)	1136 (24.9)	1712 (20.9)			
Overweight	1929 (53.0)	2103 (46.1)	2895 (35.3)			
Obese	825 (22.6)	847 (18.5)	1020 (12.4)			
**CVD diagnosis**						
No	1388 (38.1)	4169 (91.5)	5536 (67.6)	198.197	1	< 0.0001
Yes	2248 (61.9)	386 (8.5)	2634 (32.2)			
**Dyslipidemia**						
No	2249 (61.8)	2845 (62.4)	5094 (62.1)	1144.317	1	< 0.0001
Yes	1387 (38.1)	1710 (37.5)	3097 (37.8)			

*df* degrees freedom, *CVD* cardiovascular disease.*Pearson X^2^ test. ** T test

Table 2 shows the distribution of the characteristics of access to health systems and actions for the control and prevention of diabetes and its complications in Brazil and Mexico. In Mexico, more patients have access to medical care than in Brazil (*p* ≤ 0.0001). In both countries, most of the participants received health care in a public system (*p* ≤ 0.0001); however, in Brazil, people have more access to private health care than in Mexico (*p* ≤ 0.001). During the national surveys, the interviewers asked about the kind of treatment used for their diabetes control. Oral medications as exclusive treatment were most frequently mentioned in both countries (*p* ≤ 0.0001). There was a significant difference between Mexico and Brazil related to the use of oral antidiabetics, being used more frequently by Mexicans (*p* ≤ 0.001). Compared to Mexico, more participants in Brazil mentioned not using any medication for glucose control, whether that be insulin, pills or both.

**Table 2:** Healthcare characteristics of diabetes participants in Brazil and Mexico surveys

	Countries					
	Brazil n (%)	Mexico n (%)	Total n (%)	***X***^***2***^	***df***	P*
**Access to medical care**						
No	931 (25.6)	267 (5.8)	1198 (14.6)	182.551	1	< 0.0001
Yes	2705 (74.3)	4288 (94.1)	6993 (85.3)			
**Type of medical care**						
Private	768 (21.1)	680 (14.9)	1448 (17.6)	184.233	3	< 0.0001
Public	1915 (52.6)	3371 (74.0)	5286 (64.5)			
Do not receive	931 (25.6)	267 (5.8)	1198 (14.6)			
Other	22 (0.6)	237 (5.2)	259 (3.1)			
**Type of diabetes treatment**						
None	742 (20.4)	600 (13.1)	1306 (15.9)	229.920	3	< 0.0001
Insulin	162 (4.4)	339 (7.4)	401 (4.8)			
Oral medications	2253 (61.9)	3397 (74.5)	5650 (68.9)			
Both	479 (13.1)	290 (6.3)	769 (9.3)			
**Measurement of venous glucose**						
No	717 (19.7)	2707 (59.4)	3424 (41.8)	1474.135	1	< 0.0001
Yes	2919 (80.2)	1848 (40.5)	4767 (58.2)			
**Use of glucose strips (glucometer)**						
No	1631 (44.8)	3238 (71.0)	4869 (59.4)	914.141	1	< 0.0001
Yes	2005 (55.1)	1317 (28.9)	3322 (40.6)			
**Hb**_**A1C**_						
No	1403 (38.5)	3654 (80.2)	5057 (61.7)	3126.558	1	< 0.0001
Yes	2233 (61.4)	901 (19.7)	3134 (38.2)			
**Urinalysis**						
No	1102 (30.3)	2675 (58.7)	3777 (46.2)	1618.638	1	< 0.0001
Yes	2534 (69.6)	1880 (41.3)	4414 (53.8)			
**Foot exam**						
No	1821 (50.0)	3395 (74.6)	5216 (63.7)	1661.402	1	< 0.0001
Yes	1815 (49.9)	1161 (25.4)	2976 (36.3)			

*df* degrees freedom, *X^2^ Test

Relating to indicators of glycemic control, more participants in Brazil reported measuring venous glucose and Hb_A1C_ and using a capillary glucose (glucometer). As a preventive action, more people in Brazil performed urinalysis (*p* ≤ 0.001) and foot exams (*p* ≤ 0.001).

### Principal diabetic complications

The most prevalent complication was visual impairment in both countries (*p* ≤ 0.0001). More participants reported visual alterations due to diabetes in Mexico than in Brazil (*p* ≤ 0.001). Leg ulcers were the second most common complication associated with diabetes mentioned by people in both countries. The remaining complications evaluated did not reveal differences between the interviewed populations. Importantly, nearly 50% of participants developed one or more complication.

### Logistic regression

After the selection of variables, the total participants with and without complications were 4198 and 3993, respectively. In the simple logistic models, each variable with significant differences between the countries was tested for its association with diabetic complications (Table 3). In these models, Mexico had twice the probability of having a complication (*p* ≤ 0.0001). Comparing those participants living in metropolitan areas with those in interior and rural homes, rural dwellers had higher odds of complications (11, and 21%, respectively). Sex and Hb_A1C_ were not significant in either the simple or adjusted model. However, we observed a statistical trend (*p* = 0.07) in sex in the adjusted model, where women presented as having lower odds (10%) than men for having a diabetic complication. When adjusting for other variables (area of residence, time living with diabetes, type of medical care, type of diabetes treatment, measure of venous glucose, use of glucose strips, urinalysis, foot exam, diagnosis of cardiovascular disease and dyslipidemia), participants from Mexico were three times more likely to present complications from diabetes. The time with diabetes was a significant predictor of complications. Participants with a history of diabetes between 5 and less than 10 years had a 39% greater chance of having a complication than those with less than 5 years. The odds for those between 10 and 15 years were 94%, and the odds for those with 15 years or more were 147%.

**Table 3:** Regression analysis and multiple logistic predictions for diabetic complications in adults

Term	Simple model		Adjusted model	
	OR (95% IC)	***P***-Value	OR (95% IC)	***P***-Value
Country (reference: Brazil)				
Mexico	2.01 (1.84–2.20)	< 0.0001	3.04 (2.54–3.63)	< 0.0001
Area of residence (reference: Urban)				
Rural	1.22 (1.09–1.36)	0.0004	1.22 (1.06–1.39)	0.0049
Sex (reference: Men)				
Women	1.03 (0.94–1.12)	0.5707	0.90 (0.80–1.01)	0.0791
Age (years)	1.01 (1.01–1.01)	< 0.0001	1.00 (0.99–1.01)	0.4822
Time living with diabetes (reference < 5 years)				
5–10 years	1.52 (1.34–1.71)	< 0.0001	1.39 (1.21–1.61)	< 0.0001
10–15 years	2.11 (1.85–2.40)	< 0.0001	1.94 (1.66–2.26)	< 0.0001
15 years or more	2.72 (2.42–3.06)	< 0.0001	2.47 (2.11–2.89)	< 0.0001
Type of Medical care (reference: Private)				
Public	1.56 (1.38–1.76)	< 0.0001	1.31 (1.13–1.52)	0.0003
Other	2.32 (0.99–5.47)	0.0538	3.13 (1.11–8.89)	0.0316
No medical care	0.53 (0.45–0.62)	< 0.0001	0.93 (0.76–1.14)	0.4939
Type of diabetes treatment (reference: None)				
Oral medications	2.06 (1.80–2.35)	< 0.0001	1.20 (1.01–1.43)	0.0364
Insulin	4.02 (3.18–5.08)	< 0.0001	1.67 (1.25–2.24)	0.0005
Both	4.60 (3.80–5.56)	< 0.0001	2.19 (1.70–2.81)	< 0.0001
Silhouette (reference: normal weight)				
Underweight	1.78 (1.50–2.12)	< 0.0001	1.46 (1.21–1.75)	0.0001
Overweight	0.98 (0.87–1.11)	0.7846	1.00 (0.88–1.13)	0.9676
Obese	0.77 (0.66–0.90)	0.001	1.05 (0.87–1.26)	0.6146
Physical activity (reference: No)				
Yes	0.65 (0.57–0.73)	< 0.0001	0.72 (0.62–0.85)	< 0.0001
Alcohol consumption (reference: No)				
At least monthly	0.68 (0.58–0.78)	< 0.0001	0.85 (0.71–1.01)	0.0656
Less than monthly	0.91 (0.81–1.02)	0.1171	0.84 (0.74–0.97)	0.0162
Measurement of venous glucose (reference: No)				
Yes	1.48 (1.35–1.63)	< 0.0001	1.53 (1.33–1.76)	< 0.0001
Use of glucose strips (glucometer) (reference: No)				
Yes	1.27 (1.16–1.39)	< 0.0001	1.37 (1.21–1.55)	< 0.0001
HbA1C (reference: No)				
Yes	0.93 (0.84–1.02)	0.1095	0.99 (0.83–1.17)	0.9046
Urinalysis (reference: No)				
Yes	1.35 (1.24–1.47)	< 0.0001	1.28 (1.12–1.47)	0.0004
Foot exam (reference: No)				
Yes	1.22 (1.11–1.35)	< 0.0001	1.26 (1.09–1.46)	0.0020
Cardiovascular disease diagnosis (reference: No)				
Yes	1.19 (1.09–1.3)	0.0001	1.16 (1.04–1.30)	0.0086
Dyslipidemia diagnosis (reference: No)				
Yes	1.40 (1.27–1.55)	< 0.0001	1.54 (1.36–1.73)	< 0.0001
Don’t know	1.45 (1.27–1.65)	< 0.0001	1.29 (1.10–1.51)	0.0021

Participants that had private health care; were using any type of medication; who performed blood strip testing, urinalysis, serum blood glucose measurements, and foot exams; were underweight; or had a history of cardiovascular and dyslipidemia had a significantly greater chance of having diabetes complications even in the adjusted model.

## Discussion

This article aimed to compare the prevalence of diabetes complications in Brazil and Mexico using two official national surveys. The data collected in both the National Health Survey (PNS) of Brazil and the National Survey of Health and Nutrition (ENSANUT) in Mexico had several similarities. Both are public health instruments with the objective of collecting information about the population in different regions and represent the most important epidemiological information available about the population’s health.

The prevalence of diabetes complications was higher in Mexico than in Brazil. This result could be explained from different perspectives; however, the most important factor was the time living with diabetes after diagnosis. More than 15 years living with diabetes conferred a two times greater risk of developing a complication, most commonly retinopathy. In our study in Mexico, more people reported more than 15 years since diagnosis than in Brazil. Another important aspect was the genetic variants in both populations [15, 16]. In the Mexican population, some specific genotypes conferred a higher risk of developing diabetes and its complications [17]. Although these two Latin American countries have different social structures, Mexico has a greater degree of miscegenation based on autochthonous indigenous populations [18], and Brazil has a higher proportion of Afro-descendants and a lower indigenous component [19]; these genetic factors affect the risks of developing metabolic diseases [20, 21].

Older age was associated with a higher odds of complications in the simple model but lost its significance when adjusting for length of diabetes. This finding may indicate that the duration of the disease is a major risk factor for diabetes complications, more so than age. In the study by Al-Saeed et al., Australian patients with an earlier onset of diabetes had an increased risk of renal and peripheral nerve complications and higher standardized mortality than those whose onset was middle age and older-adult stage [22]. The frequency of diabetes complications was similar in both sexes and countries. In a recent publication, Gedebjerg et al. [23] observed no sex differences in microvascular complications of diabetes. However, they did find a higher frequency of macrovascular complications of diabetes in men. The evidence about this item is inconclusive, but other authors have mentioned that the principal factors in the development of complications are BMI and age rather than inherent sex-related factors. In our study, BMI and age were similar in both sexes [24].

Bos et al. [25] observed that the socioeconomic and health conditions in Brazilian rural older-adults were associated with poor glucose control relative to those living in an urban environment. Our results showed that the odds of having diabetes complications were higher in those participants living in rural areas, independently of the country of the origin, age, physical activity, health access and medical treatment. However, more people live in rural areas in Mexico than in Brazil. Some factors that have been associated with this result in rural areas are a low income, low education level, migration, and health habits, such as the use of sugar and salt [26]. In this context, we observed a negative relationship between living in urban areas and physical exercise (data not shown). Lack of exercise is a known risk factor for developing diabetes and other NCDs. Moreover, it is linked to poor glucose control, which is a major risk factor for developing diabetic complications [27–29].

Compared to Brazilians, Mexicans exercise less. Physical activity, aerobic exercise specifically, has different benefits in patients with diabetes. It can improve insulin resistance and diminish hyperglycemia, therefore delaying the onset of complications [27]. Moreover, aerobic exercise can modify inflammatory and metabolic pathways that participate in macro and microvascular diabetic complications [28, 29]. This confirms the results of the present study, which found physical exercise to be a protective factor against developing diabetic complications.

In the present study, we observed that the participants who were underweight had a greater risk of diabetes complications. In Mexico, more people were underweight than in Brazil. A cohort study conducted by Sairenchi et al. [30] in Japan found that a lower BMI in older-adults 60 to 79 years old was associated with a higher risk of developing diabetic complications. Some authors have tried to explain this phenomenon by the fact that lean patients with diabetes have an accelerated loss of pancreatic beta-cells and poorer glycemic control [31]. However, this pathophysiological paradigm continues to be unanswered.

Access to a health care system plays an important role in the development of complications. In Brazil, more people have access to private medical care than in Mexico. However, in Mexico, people have more access to medical care in general. Some authors have reported a higher risk of developing retinopathy and nephropathy in people that use public healthcare systems. This relation is sustained by socioeconomic disparities because most people that use public services have a low income and therefore have less access to medicines and novel therapies. Moreover, low education level and limited access to diagnostic tools are other contributing factors [30–32].

Our findings showed a significant increase in the risk of complications according to treatment. However, the use of insulin or oral antidiabetics per se did not have a direct relationship with the presence of complications [33]. In Mexico and Brazil, certain drugs are not available in the public health system due to their high cost, despite some medications being subsidized by the government [34–37]. In our study, this was a limitation because neither national health survey distinguished between groups of medications. Another factor that could contribute to this relationship is adherence to treatment. Only 37% of the patients who used insulin in combination with an oral antidiabetic agent had high compliance [36, 37]. However, several other factors, such as visual impairments, education level, and polypharmacy, affect adherence to treatment [38–40].

In the present study, the consumption of alcohol was higher in Mexicans than in Brazilians, and its consumption at least monthly was protective against diabetic complications. The scientific literature indeed has shown that moderate alcohol consumption has been consistently associated with a decreased risk of type 2 diabetes compared to abstention or excessive consumption [41–43]. However, we could not distinguish between the kinds of alcohol (e.g., beer or red wine) or other potential confounders that might have affected our results.

Finally, visual impairment was the most frequent complication in both countries, being more prevalent in Mexico. However, because the results came from patient interviews, it was difficult to determine the exact diagnosis; the questions about visual alterations were not specific in either survey. Nonetheless, visual impairment in people with diabetes is generally (64% of the time) a consequence of diabetic retinopathy [44].

A limitation of the present study was a lack of clarity over whether the patients questioned were indigenous or Afro-descendants because the risk of developing diabetes and its complications are different in these two groups. Moreover, our study was unable to show exact information related to diagnosis, glucose control, treatment, how often people undertook prevention measures and differences between the populations. However, this first approach allowed us to observe similarities and differences between these two countries with the largest populations in Latin America.

## Conclusion

Diabetes complications are important health problems in Brazil and Mexico. Visual impairment was the principal complication in both countries, being more prevalent in Mexico. Several factors, such as access to and type of health system, living in rural areas, treatment, BMI and performing preventive actions, increased the risk of developing a complication. However, having lived with diabetes for more than 15 years was the principal risk factor. National health surveys add significant information about the impact of diabetes on these Latin American populations. This comparison of data could provide valuable information to guide national policies and program decisions in both countries.

## Data Availability

The data that support the findings of this study are free available from at the Brazilian Institute of Geography and Statistics with the Ministry of Health/Brazil (https://biblioteca.ibge.gov.br/visualizacao/livros/liv91110.pdf.) and the National Institute of Public Health/Mexico (https://ensanut.insp.mx/encuestas/ensanut2018/descargas.php) respectively. All data generated or analyzed during this study are included in this published article.

## Abbreviations

BMI: Body Mass Index
ENSANUT: National Survey of Health and Nutrition (Mexico)
IDF: International Diabetes Federation
NCDs: Non-communicable Diseases
OR: Odds ratio
PNS: National Health Survey (Brazil)
